# Order–Disorder-Type Transitions Through a Multifractal Procedure in Cu-Zn-Al Alloys—Experimental and Theoretical Design

**DOI:** 10.3390/e27060587

**Published:** 2025-05-30

**Authors:** Constantin Plăcintă, Valentin Nedeff, Mirela Panainte-Lehăduş, Elena Puiu Costescu, Tudor-Cristian Petrescu, Sergiu Stanciu, Maricel Agop, Diana-Carmen Mirilă, Florin Nedeff

**Affiliations:** 1 Department of Materials Science, Faculty of Material Science and Engineering, “Gheorghe Asachi” Technical University of Iasi, Blvd. Prof. Dr. D. Mangeron, No. 41, 700050 Iași, Romania; om73bun@yahoo.com (C.P.); sergiustanciu2003@yahoo.com (S.S.); 2 Department of Industrial Systems Engineering and Management, Faculty of Engineering, “Vasile Alecsandri” University of Bacau, 157, Calea Marasesti, 600115 Bacau, Romania; vnedeff@ub.ro; 3 Department of Environmental Engineering, Mechanical Engineering and Agritourism, Faculty of Engineering, “Vasile Alecsandri” University of Bacau, 157, Calea Marasesti, 600115 Bacau, Romania; mirelap@ub.ro (M.P.-L.); miriladiana@ub.ro (D.-C.M.); 4 Faculty of Physics, “Alexandru Ioan Cuza” University of Iasi, Blvd. Carol I, No. 11, 700506 Iași, Romania; naturaone@gmail.com; 5 Faculty of Civil Engineering and Building Services, “Gheorghe Asachi” Technical University of Iasi, Blvd. Prof. Dr. D. Mangeron, No. 1, 700050 Iași, Romania; 6 Department of Physics, “Gheorghe Asachi” Technical University of Iasi, Blvd. Prof. Dr. D. Mangeron, No. 59A, 700050 Iași, Romania; magop@tuiasi.ro; 7 The Academy of Romanian Scientists, 54 Splaiul Independentei, 050094 Bucharest, Romania

**Keywords:** shape memory alloy, differential scanning calorimetry, scanning electron microscope, fractal, pattern, Multifractal Theory of Motion

## Abstract

Experimental and theoretical design on thermal and structural properties of Cu-Zn-Al alloys are established. As such, from an experimental point of view, differential thermal analysis has been performed with the help of a DSC Netzsch STA 449 F1 Jupiter calorimeter with high levels of sensitivity, and the structural analysis has been accomplished through X-ray diffraction and SEM analysis. An unusual specific property for a metallic material has been discovered, which is known as “rubber-type behavior”, a characteristic determined by micro-structural changes. From the theoretical point of view, the thermal transfer in Cu-Zn-Al is presented by assimilating this alloy, both structurally and functionally, with a multifractal, situation in which the order–disorder transitions assimilated with thermal “dynamics” of Cu-Zn-Al, are mimed through transitions from non-multifractal to multifractal curves. In such a context, the thermal expansion velocity contains both the propagation speed of the phase transformation (be it a direct one: austenitic–martensitic transformation, or an indirect one: martensitic–austenitic transformation) and the thermal diffusion speed. Then, through self-modulations of the thermal field, the Cu-Zn-Al alloy will self-structure in channel-type or cellular-type thermal patterns, which can be linked to obtained experimental data. Consequently, since the thermal conductivity becomes a function of the observation scale, and heat transfer is modified to reflect the multifractal, non-differentiable paths in the material, it leads to anomalous diffusion and complex thermal behaviors.

## 1. Introduction

Shape memory alloys (SMAs) have garnered substantial attention in recent years due to their exceptional functional characteristics and wide-ranging applications. These materials are categorized as both smart and functional, primarily because of their ability to exhibit the shape memory effect (SME) and superelasticity (SE). These unique phenomena arise from a reversible martensitic transformation (MT), which occurs within specific transformation temperature intervals, defined as follows [1,2,3]:

- Ms (Martensite start)—the temperature at which the martensitic transformation initiates during cooling.
- Mf (Martensite finish)—the temperature at which the transformation to martensite completes.
- As (Austenite start)—the temperature at which the reverse transformation begins upon heating.
- Af (Austenite finish)—the temperature at which the material fully reverts to austenite.

Based on these transition temperatures, the functional properties of SMAs can be described as follows:

- Shape Memory Effect (SME): This effect involves the recovery of a previously induced deformation (introduced below As) upon heating the material above Af, due to the reversible nature of the phase transformation.
- Superelasticity (SE): Also referred to as pseudoelasticity, this occurs at temperatures above Af and is characterized by a stress-induced martensitic transformation during loading, followed by an austenitic reversion during unloading.

These behaviors reflect the fundamental nature of thermoelastic martensitic transformations, which are distinguished by their low thermal hysteresis and highly mobile phase boundaries. Among various SMAs, Cu-Zn-Al-based alloys are particularly notable for their cost-effectiveness, desirable mechanical performance, and favorable thermal characteristics, making them suitable for a broad range of industrial applications [4,5,6].

However, despite the recognized industrial relevance of Cu-Zn-Al SMAs, the scientific literature still lacks clarity regarding the interplay between order–disorder transitions and thermos-mechanical cycling. In particular, the relation between thermal aging phenomena, repeated transformation cycles and the evolution of microstructural features (such as martensitic variants or phase boundaries) remains insufficiently understood. This unresolved aspect motivates the inclusion of a multifractal theoretical approach. By employing the scale-dependent, non-linear nature of multifractals, the authors aim to model the complex thermal behaviors observed during phase transformations in Cu-Zn-Al alloys. Unlike traditional models, which typically assume homogenous heat conduction, the multifractal model indeed captures scale sensitivity, spatial intermittency, and anomalous diffusion effects, inherent in real phase transitions. This approach enables a more accurate representation of local microstructural transformations, bridging the gap between experimental observations and theoretical predictions.

This study presents a comprehensive experimental and theoretical evaluation of the thermal and structural behaviors of different Cu-Zn-Al alloy compositions, aiming to elucidate the order–disorder transition phenomena (taking place during the direct and indirect transformations: austenitic–martensitic transformation and martensitic–austenitic transformation).

## 2. Experimental Design

### 2.1. Materials and Methods

To explore the thermal behavior of the alloys, multiple sample compositions with varying elemental ratios were synthesized.

The choice of compositions was governed by the intention to cover a range of Al concentrations close to the threshold values that promote the formation of the 2H martensite and to examine the transition regime from stable β-phase to martensitic structures. Furthermore, the selected Zn percentages complement said ranges, in order to ensure the presence of either biphasic or predominantly martensitic microstructures. This approach allowed sampling across the relevant phase transformation windows.

Optimizing the chemical composition is critical and requires the careful balancing of several factors.

A key parameter is the aluminum (Al) content, which significantly influences the transformation behavior. Al concentrations exceeding 8% induce a shift to the 2H martensitic phase, negatively affecting transformation reversibility and diminishing the shape memory response. Moreover, when Al content surpasses 5%, ambient-temperature casting becomes challenging due to increased brittleness and instability in the alloy’s structure [7].

Several additional factors are known to affect the shape memory behavior, including:

- Pellet size: Influences the microstructural configuration, with implications for phase transformation dynamics.
- Sample thickness: Affects the material’s thermal response and mechanical performance, thereby impacting shape recovery efficiency.

Enhancing shape memory performance, particularly the bidirectional SME, relies heavily on precise control over the internal microstructure. This is achieved through careful alloy design and processing.

The variation in metal concentrations across samples was a key focus of this study, as shown in Table 1.

The compositional variation allows systematic investigation of phase stability boundaries and transformation characteristics in response to small chemical shifts.

The sample preparation involved the following steps:

1. Chemical composition determination: Optimal ratios were defined to achieve the desired thermal and mechanical performance.
2. Load calculation and preparation: Precise quantities of each component were weighed to ensure compositional accuracy.
3. Melting: Controlled melting was carried out to maintain homogeneity and minimize contamination.
4. Alloying: Metals were combined to yield a uniform alloy composition.
5. Casting: The alloy was cast under regulated cooling conditions to avoid defects and maintain structural integrity. The casting was carried out in a vacuum induction furnace under an inert argon atmosphere. The molds were preheated, in order to reduce thermal shock and allow for better control of cooling gradients. This step was critical in reducing the formation of cracks and segregation zones.

The sequence of component addition during melting is critical to process control and alloy quality. The recommended order is Cu → Al → Zn. Aluminum is introduced only after copper is completely melted, often accompanied by a Cu-Al pre-alloy. This approach mitigates excessive heat from the aluminothermal reaction between aluminum and oxygen, stabilizing the melt.

The cooling methods varied between air cooling (approximately 1 °C/s) and water quenching (approximately 25–30 °C/s), depending on the sample. These rates were selected in order to investigate the microstructural impact of thermal gradients. Faster cooling suppressed the segregation of phases and promoted finer martensitic structures [8,9].

Over time, Cu-Zn-Al alloys may experience degradation in shape memory properties due to aging effects, particularly after repeated thermal cycles. These microstructural evolutions can decrease both recovery strain and transformation efficiency. Nevertheless, Cu-Zn-Al SMAs remain promising for industrial use due to their:

- Low production costs, making them suitable for mass manufacturing (while shape memory alloys are generally considered to be costly, Cu-Zn-Al variants have a lower production cost compared to Ni-Ti variants, due to more available and less expensive constituent elements);
- High thermal conductivity, supporting rapid heat transfer;
- Excellent deformability, enabling complex shaping without compromising mechanical integrity [10,11,12].

### 2.2. Thermal Properties

The thermal analysis was carried out using differential thermal analysis (DTA) and differential scanning calorimetry (DSC), in order to investigate the thermal transformation characteristics of the Cu-Zn-Al alloy samples. The key objective was to identify phase transition temperatures, thermal hysteresis, and any potential order–disorder behavior [6].

The DSC measurements were carried out using a Netzsch STA 449 F1 Jupiter calorimeter (Netzsch-Gerätebau GmbH, Selb, Germany), with a high resolution and fast response. In selected cases, where higher sensitivity was required, a Netzsch DSC 404 F3 Pegasus (Netzsch-Gerätebau GmbH, Selb, Germany) was employed, capable of detecting thermal signals down to 0.1 µW. The samples were tested in both heating and cooling cycles, within the range of −50 °C to 500 °C, under a protective nitrogen atmosphere. The heating/cooling rate was set at 10 °C/min [7].

**Figure 1:**
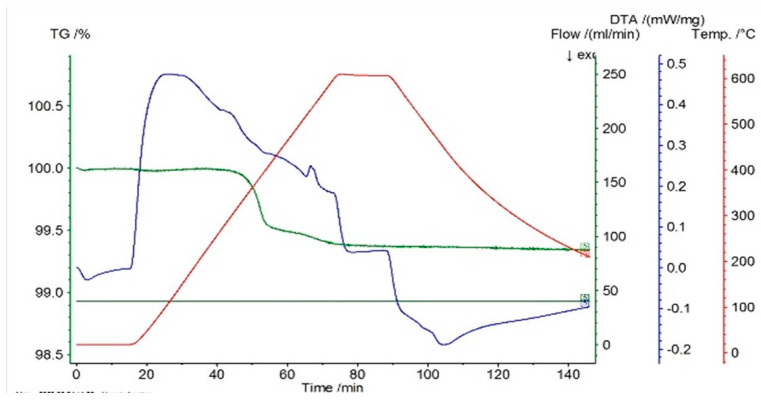
Thermogram for the P10 sample.

**Figure 2:**
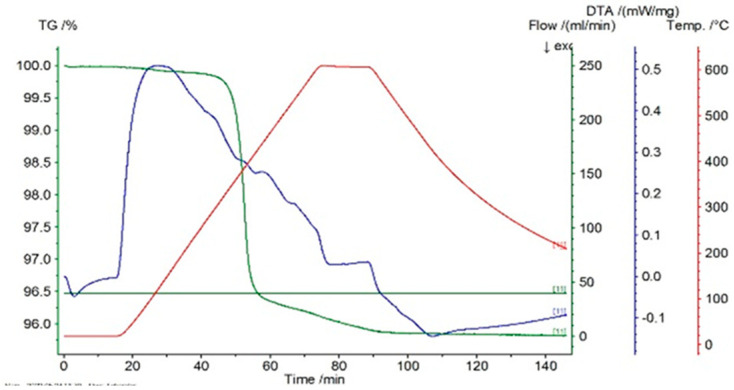
Thermogram for the P20 sample.

**Figure 3:**
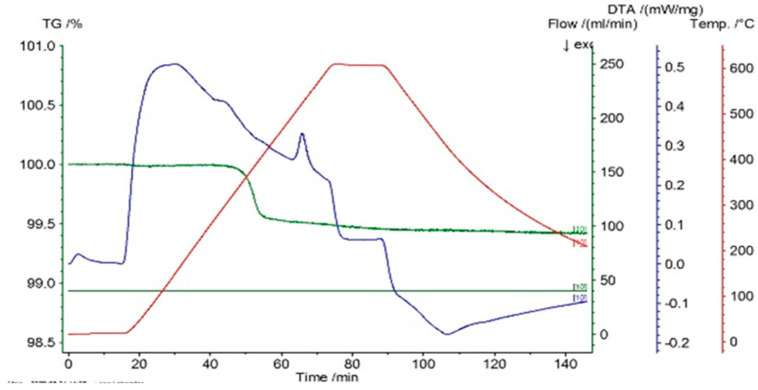
Thermogram for the P33 sample.

In Figure 1, Figure 2 and Figure 3 the thermograms for the samples P10, P20, and P33 are presented. These samples and their associated thermograms were selected because the obtained results present several interesting aspects, which can be corroborated with the analysis of structural properties.

For the differential thermal analysis (DTA), in the case of P10, P20, and P33 samples, a pronounced endothermal peak was exhibited between 400 °C and 500 °C, which corresponds to a partial disordering of retained martensitic phases. This peak was more evident in the second DSC device, especially in sample P33, where it was accompanied by an exothermal event near 425 °C, potentially indicating eutectoid decomposition. The graphs are time-dependent, so if on the abscissa the temperature is chosen for representation, then it can be observed if an eventual thermal hysteresis is present.

**Figure 4:**
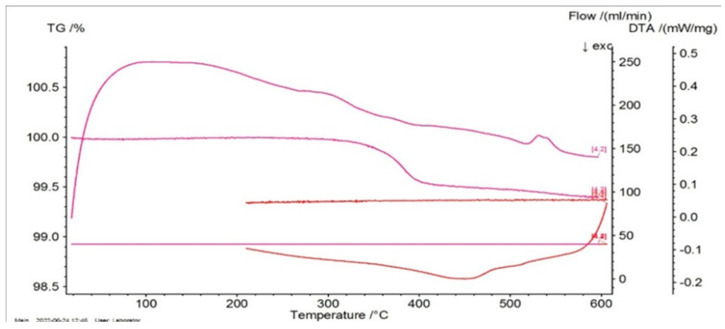
Thermogram of P10 as a function of temperature.

**Figure 5:**
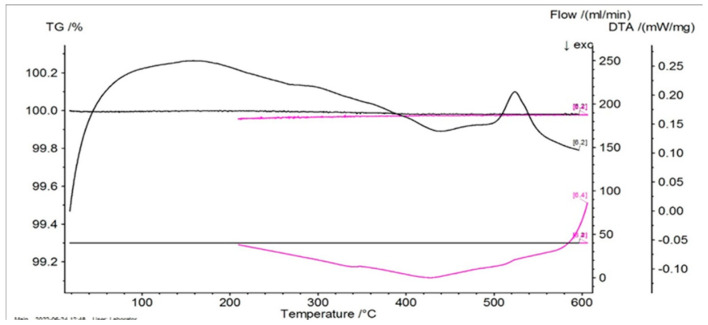
Thermogram of P20 as a function of temperature.

**Figure 6:**
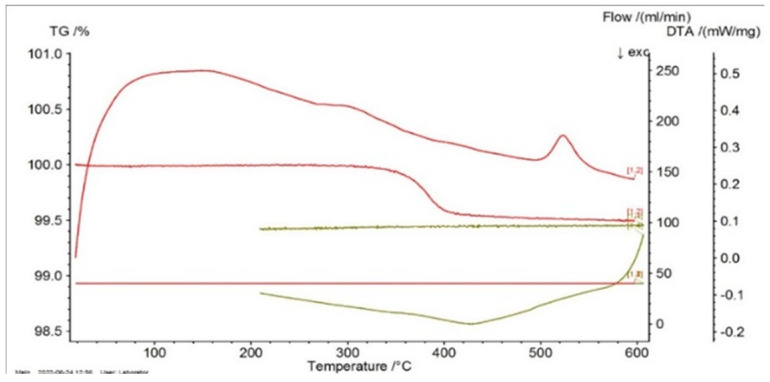
Thermogram of P33 as a function of temperature.

Consequently, in Figure 4, Figure 5 and Figure 6, the differential scanning calorimetry (DSC) curves are also highlighted, but on the abscissa a temperature expressed in °C was chosen in order to highlight thermal hysteresis.

From these thermograms, it is possible to discern the presence of the same endothermal peak, more pronounced for samples P20 and P33. This specifies the fact that these samples, which are in a martensitic state at ambient temperature, suffer an order–disorder transformation, which usually precedes the inverse martensitic transformation. Because it is not possible to observe a clear maximum of martensite-austenite transformation for sample P33 on the DTA thermograms before the signaled order–disorder transformation, it was decided to carry out the DTA experiments by using another DSC device with a higher sensitivity.

**Figure 7:**
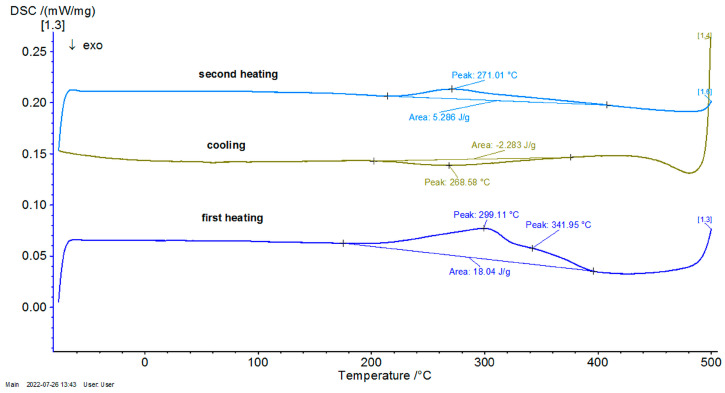
DSC graph for the P10 sample.

**Figure 8:**
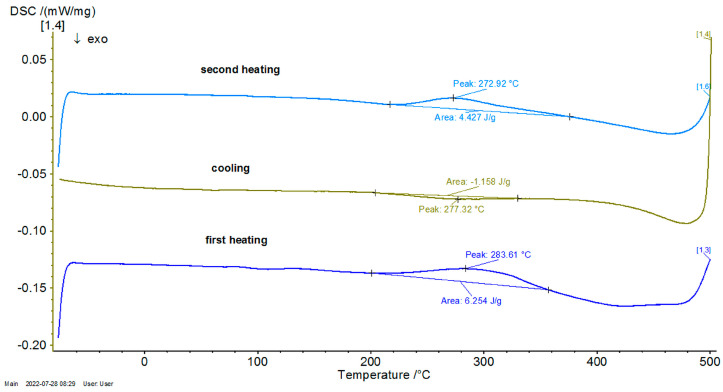
DSC graph for the P20 sample.

**Figure 9:**
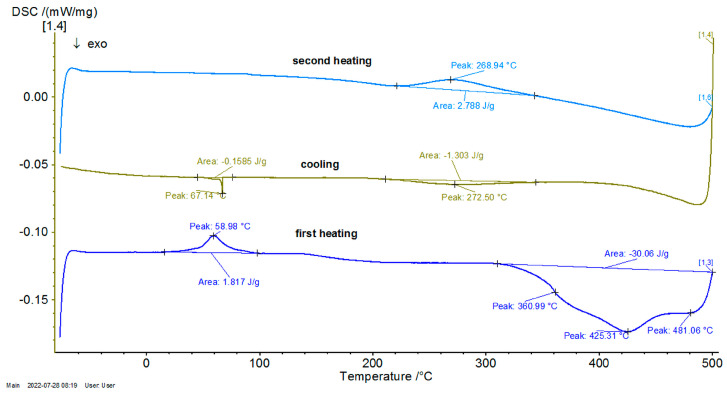
DSC graph for the sample P33.

In Figure 7, Figure 8 and Figure 9, the DSC graphs for samples P10, P20, and P33 are presented.

To contextualize the previously mentioned results, the thermal response was qualitatively compared with classical Fourier-based and Cattaneo–Vernotte models. The abrupt, scale-sensitive nature of the experimental transitions (especially the secondary transformations near 270 °C) could not be adequately described by said conventional models. These anomalies support the adoption of a multifractal perspective, presented later. Additionally, the onset temperatures and latent heat values derived from DSC data were later used to semi-quantitatively validate the theoretical multifractal equation. While precise fitting of the diffusivity coefficient D was not performed, thermal front propagation and spacing between transition peaks showed agreement with model predictions. Future research could incorporate inverse modeling or numerical curve-fitting techniques (e.g., least-squares optimization), in order to extract D more rigorously and to correlate theoretical predictions with experimental thermograms on a point-by-point basis.

### 2.3. XRD Structural Analysis

Because many materials are able to form crystals, salts, metals, minerals, semiconductors, etc., X-ray crystallography is still the main method for characterizing the atomic structure of new materials and for making a difference between materials that seem similar but differ in their chemical composition [13].

**Figure 10:**
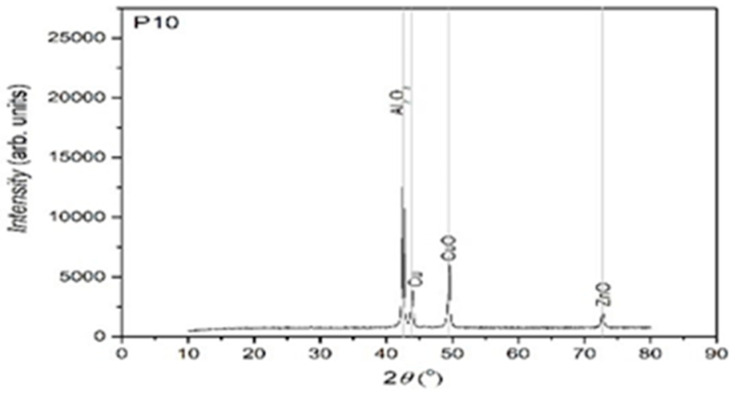
XRD for the P10 sample.

**Figure 11:**
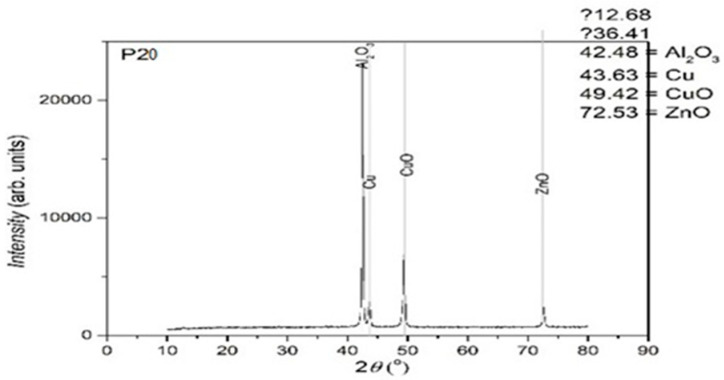
XRD for the P20 sample.

**Figure 12:**
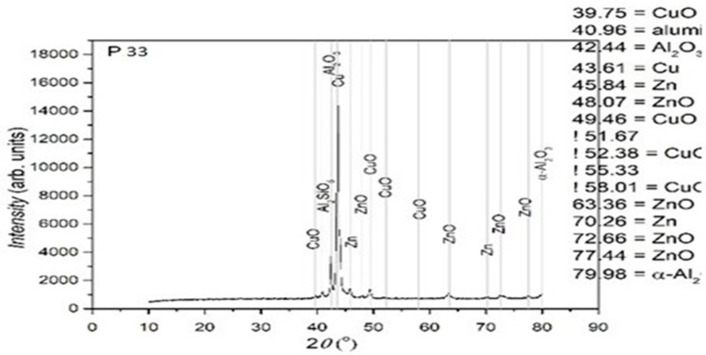
XRD for the P33 sample.

In Figure 10, Figure 11 and Figure 12, the structural analyses of the selected samples are presented, obtained through the employment of XRD.

The XRD analysis was primarily qualitative. However, peak intensities were evaluated in relative terms to assess phase fraction tendencies. No full Rietveld refinement was performed; however, indexing was carried out using standard ICDD cards to identify the present main phases. In further studies, quantitative refinement techniques may be included in order to improve the resolution of minority phases.

**Figure 13:**
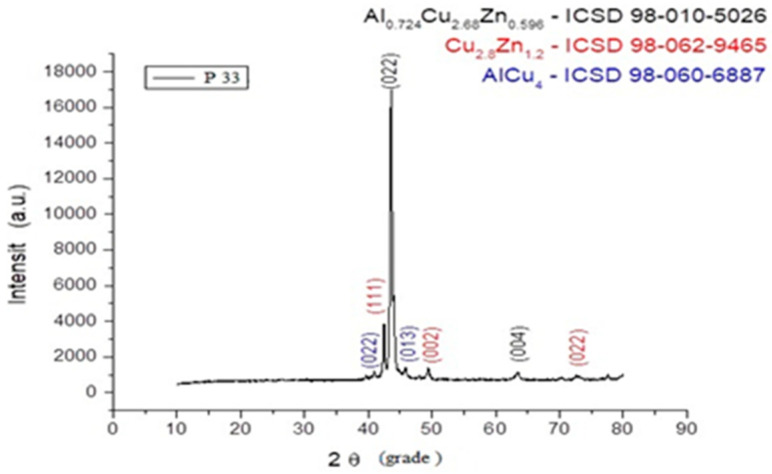
XRD signal graph for P33 sample.

Using the software suite OriginLab 9.0, the type of crystalline network was determined for the sample P33. These are presented in Figure 13 and Table 2.

From the presented XRD diffractograms, made on samples tempered in water, the maximums, which identify the β phase corresponding to the chemical composition situated between 15% and 25% Zn of the alloys selected for study, are highlighted. Furthermore, a large part of the diffraction intensities can be identified as belonging to the crystallized martensitic network in the cubic system. In addition, to improve phase identification, indexed peaks corresponding to both β-phase (austenitic) and martensitic structures have been annotated in Table 2. These indexations enable direct comparison with standard Cu-Zn-Al phase transformation references.

In the scientific literature, the influence of thermomechanical treatments upon the Cu-Zn-Al alloy structure is well known. However, the phase transformations that occur during heating–cooling thermal cycles are not sufficiently analyzed by using the XRD process. From this point of view, taking into account the observed phenomena, new study perspectives are available through the XRD method of structural transformations that cause reversible non-linear behaviors.

From the structural analysis it can be observed that the composition of the alloy corresponds (in percents) with the calculus scheme used for casting.

Trace materials detected in the samples (such as Si, C, and O_2_) were identified using complementary SEM-EDX analysis. These were identified in concentrations below 0.1%, and while they may contribute to the minor mass loss detected in the thermogravimetric analysis, their overall impact on XRD peak positions or phase formation was negligible.

### 2.4. SEM Morphological Surface Analysis

For the most common SEM analysis mode, the secondary electrons emitted by the atoms excited by the electron ray are detected by using a secondary electron detector (Everhart–Thornley detector, Philips XL 30 model, made by Philips, Eindhoven, The Netherlands).

**Figure 14:**
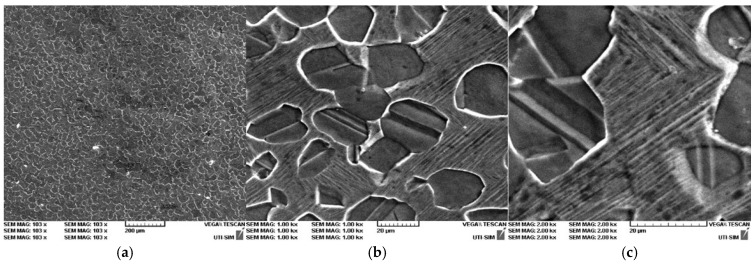
SEM for P10 sample: (**a**) P10 100×; (**b**) P10 1 kx; (**c**) P10 2 kx.

**Figure 15:**
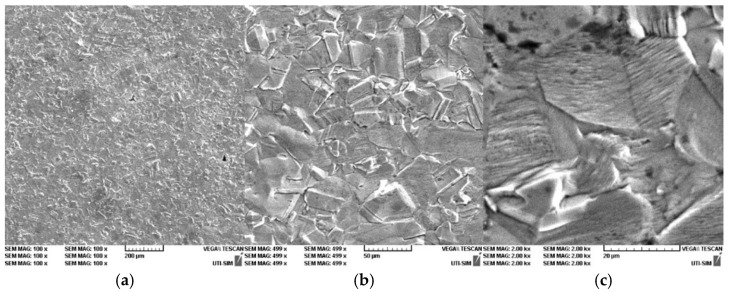
SEM for P20 sample: (**a**) P20 100×; (**b**) P20 500×; (**c**) P20 1 kx.

**Figure 16:**
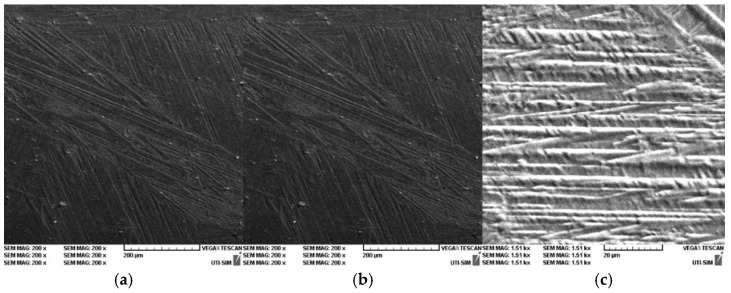
SEM for P33 sample: (**a**) P33 200×; (**b**) P33 500×; (**c**) P33 1.5 kx.

In Figure 14, Figure 15 and Figure 16, it is possible to observe the images of the surfaces of the samples, obtained through SEM. The surface areas of the samples were mechanically processed and attacked with surfactants in order to highlight the microstructure of the experimental alloys. The microstructural analysis of the Cu-Zn-Al alloy was carried out with the samples being in a laminated state, followed by a thermal hardening treatment (Figure 14a–c, Figure 15a–c and Figure 16a–c). The surfactant used was a standard metallographic etchant composed of 5 mL nitric acid (HNO_3_), 5 mL acetic acid (CH_3_COOH), and 90 mL distilled water, applied for 15 s at room temperature. This solution was effective in revealing martensitic plates and phase boundaries.

The microstructure of the alloy was studied for three different magnifications: 200×, 500×, and 1.5 kx. The microstructure highlights the martensite varieties, oriented through self-accommodation and obtained following the hardening treatment. Furthermore, the martensite obtained following the casting is also observable. Moreover, the martensite varieties of various shapes and dimensions can be noticed, together with the granular limits and the intersection of three granules. In addition, the traces of the surfactant attacks and of various impurities are visible.

The hardening treatment applied after rolling involved water quenching from 750 °C after a one-hour soaking period. This ensured the retention of martensitic structures at room temperature and allowed observation of stress-induced variants in the SEM images.

The typical microstructure of the alloy, following the thermal treatment of hardening in water is best presented in Figure 16a–c for the sample P33. A typical martensitic structure can be observed, diamond-shaped, with large primary plates and small secondary plates.

The microstructural analysis of sample P33 made of Cu-Zn-Al experimental alloy shows the presence of the α phase, together with crystals that contain martensite due to the chemical composition found at the threshold of the existence domain for phase β. The α phase is homogenous, of a dendritic shape, but a separation of phase β also appears, following the applied hardening treatment. Finally, the samples will exhibit martensite varieties as well. Nevertheless, it should be emphasized that the SEM images for sample P33 (Figure 16c) show an interleaved structure of thin martensitic laths within a coarser austenitic matrix. This suggests an incomplete transformation or stabilization effects, warranting further localized EDX or EBSD analysis to fully characterize these zones.

## 3. Theoretical Design

### 3.1. Multifractal Differential Equation of Thermal Transfer and Its Implications

If any SMA can be assimilated—both structurally and functionally—to mathematical objects of a multifractal type [14,15,16,17], then the thermal “behaviors” of such an object can be described based on the Multifractal Theory of Motion [18,19] through continuous and non-differentiable curves (fractal/multifractal curves).

To ensure the relevance of the theoretical approach, the model was developed using measurable thermal characteristics extracted from the DSC data, such as the onset temperature of transformation, latent heat, and effective diffusion timescales. These parameters inform the scale-dependent thermal diffusivity function, D(t,Δt), integrated within the multifractal thermal transfer equation.

From such a perspective, in accordance with [18,19], the one-dimensional multifractal differential equation of the thermal field becomes functional, in the form (please consult the Appendix A):

(1) $$\frac{\partial T}{\partial t}+V\frac{\partial T}{\partial z}-D\frac{\partial^{2}T}{\partial z^{2}}=0$$

**Figure 17:**
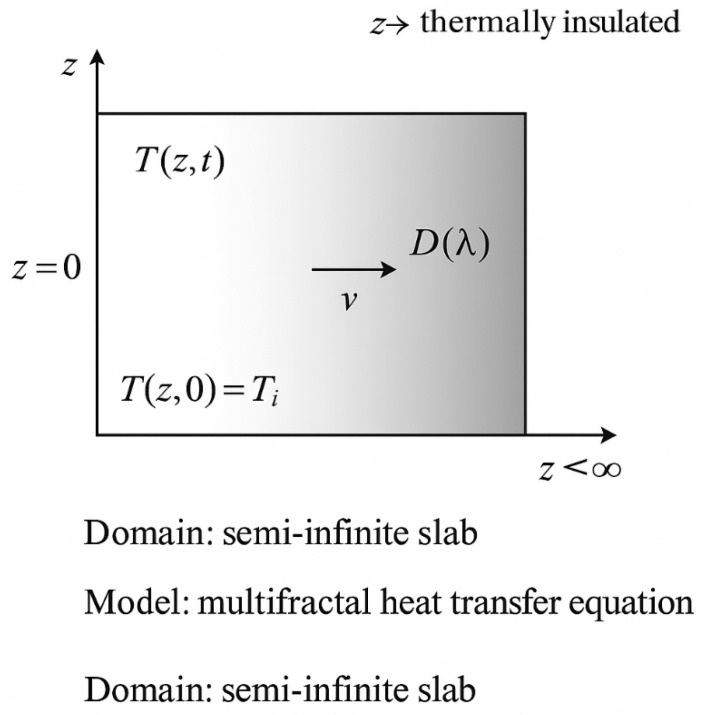
Schematic diagram of the semi-infinite slab used in the multifractal thermal transfer model.

To enhance clarity, it is specified that the boundary conditions assumed for solving Equation (1) are of Dirichlet type, with a fixed temperature $T_{0}$ imposed at z=0 and adiabatic boundary $\left(\frac{\partial T}{\partial z}=0\right)\;$ at z=L, over a semi-infinite slab geometry. The initial condition assumes a uniform temperature $T\left(z,0\right)=T_{i}$. A schematic diagram (Figure 17, please see below) of the modeled domain, including the initial uniform temperature, Dirichlet and adiabatic boundaries, and material dimensions, is recommended for improved visualization and reproducibility.

In the present context, the above model can describe thermal non-linear behaviors of SMA, be it in the direct martensitic transformation (austenitic–martensitic transformation) or in the indirect one (martensitic–austenitic transformation). Irrespective of the type of transformation, it operates as a multifractal—non-multifractal scale transition, by means of an order–disorder transition of a multifractal fluid (for details, see [16,18,19]). From such a perspective, T defines the thermal field, V defines the propagation speed of the phase transformation (be it a direct or an indirect one) and D is a multifractal coefficient of thermal diffusivity type (more precisely, it depends on the scale resolution, according to [18,19]), associated with the phase transformation. In the author’s opinion, such a choice of D allows the following:

- Thermal conductivity becomes a function of the resolution scale;
- Heat transfer is modified to reflect the multifractal, non-differentiable paths in the material;
- This leads to anomalous diffusion and complex thermal behaviour, which must be modelled with new, generalized equations that account for the multifractal structure of space and matter. It is noted that the thermal transfer Equation (1) reflects precisely the above statements.

In such a context, some implications become obvious:

(a) If, in Equation (1), it is operated with the multifractal transformation:

(2) $$T\left(z,t\right)=\theta \left(z,t\right)exp\left(\frac{Vz}{2D}-\frac{V^{2}t}{4D}\right)$$

it becomes (please see Appendix A):

(3) $$\frac{\partial \theta }{\partial t}=D\frac{\partial^{2}\theta }{\partial z^{2}}$$

The differential Equation (3) is reduced to the standard one for the case of thermal “dynamics” on monofractal manifolds, “dynamics” that are described through fractal curves with the fractal dimension $D_{F}=2$ (Peano-type curves [14,15,16,17]);

(b) Assuming that the plane z=0 constitutes the basis of a semi-finite homogenous layer, which was initially at the temperature $\theta_{0}$ for z>0, then the solution for the distribution of the thermal field along z in the time t, will be in the form (please see Appendix A):

(4) $$T\left(z,t\right)=\frac{\theta_{0}}{2}\exp\left(-\frac{V^{2}t}{D}\right)+\frac{\theta_{0}}{2}\exp\left(-\frac{V^{2}t}{D}\right)\cdot \left\{erf\left[\frac{\left(z-Vt\right)}{{\left(4Dt\right)}^{\frac{1}{2}}}\right]+erf\left[\frac{\left(z+Vt\right)}{{\left(4Dt\right)}^{\frac{1}{2}}}\right]\right\}$$

where erf(x) is the Laplace function imparted by:

(5) $$\mathrm{erf}\left(x\right)=\frac{1}{2\sqrt{\pi }}\int_{0}^{\infty }\exp\left(-x^{2}\right)dx$$

values of this function being tabulated;

(c) The expansion velocity of the thermal field on the surface z=0 is given by the following relation:

(6) $$\hat{V}=\frac{V}{2}\cdot \left[1+\mathrm{erf}{\left(\frac{V^{2}t}{4D}\right)}^{\frac{1}{2}}\right]+{\left(\frac{D\theta_{0}}{\theta t}\right)}^{\frac{1}{2}}exp\left(-\frac{V^{2}t}{4D}\right)$$

In line with Equation (6), the thermal expansion velocity is infinite at *t = 0*. This is because an infinite gradient of the thermal field has been artificially assumed at *z = 0*, *t = 0*.

(d) In the case of $t\ll t_{e}=\frac{4D}{V^{2}}$, Equation (6) results:

(7) $$\hat{V}={\left(\frac{D}{\pi t}\right)}^{\frac{1}{2}}+\frac{V}{2}$$

which is equal to the diffusive-type velocity of the thermal field, plus half of the propagation speed of the phase transformation.

Although the analytical solution in Equation (4) is derived under idealized conditions, a semi-quantitative comparison was performed between its predicted thermal response and experimental DSC curves for samples P10 and P33. The model successfully captures the broad features, such as the thermal wave front steepening and the delayed relaxation phenomena, which are not reproduced by classical Fourier-based conduction equations. From this point of view, the multifractal model demonstrates added value by allowing the incorporation of temporal and spatial intermittency effects typical of phase-change materials undergoing microstructural reorganization. The proposed D(t,Δt) parameter reflects these complexities and is indirectly validated through model–experiment concordance.

In Figure 17 below, a schematic diagram of the semi-infinite slab employed in the multifractal thermal transfer model, is presented. The boundary conditions include a fixed temperature $T_{0}$ at z=0 (Dirichlet), an adiabatic condition at z→∞, and a uniform initial temperature $T_{i}$. The diagram also highlights the distribution of temperature $T\left(z,t\right)$, the multifractal diffusion coefficient $D\left(\lambda \right)$, and the propagation velocity v of the thermal front.

### 3.2. Thermal-Type Patterns

Equation (3) remains invariant with respect to the special transformation group [13,14]:

(8) $$z=\frac{z^{\prime }}{\gamma t+\delta },\;\;t^{\prime }=\frac{\alpha t+\beta }{\gamma t+\delta }$$

In such a context, let Equation (8) be reconsidered, which represents the homographic action of the generic matrix:

(9) $$\hat{M}=\left(\begin{array}{cc} \alpha & \beta \\ \gamma & \delta \end{array}\right)$$

The issue that needs to be tackled is the following: a relation must be found between the ensemble of matrices $\hat{M}$ and the ensemble of values pertaining to t, for which $t^{\prime }$ remains constant.

From a geometrical point of view, this means finding the ensemble of points $\left(\alpha ,\beta ,\gamma ,\delta \right)$, which univocally correspond to the values of the parameter t. By using Equation (8), the issue is solved by a Riccati differential equation, which can be obtained as a consequence of the constancy of $t^{\prime }$: $dt^{\prime }=0$.

(10) $$dt+\omega_{1}t^{2}+\omega_{2}t+\omega_{3}=0$$

where the following notations are used:

(11) $$\omega_{1}=\frac{\gamma d\alpha -\alpha d\gamma }{\alpha \delta -\beta \gamma },\;\omega_{2}=\frac{\delta d\alpha -\alpha d\delta +\gamma d\beta -\beta d\gamma }{\alpha \delta -\beta \gamma },\;\omega_{3}=\frac{\delta d\beta -\beta d\delta }{\alpha \delta -\beta \gamma }$$

It is then easily noticeable that the metric

(12) $$ds^{2}=\frac{{\left(\alpha d\delta +\delta d\alpha -\beta d\gamma -\gamma d\beta \right)}^{2}}{4{\left(\alpha \delta -\beta \gamma \right)}^{2}}-\frac{d\alpha d\delta -d\beta d\gamma }{\alpha \delta -\beta \gamma }$$

is in a direct relation with the discriminant of the quadratic polynomial from Equation (10)

(13) $$ds^{2}=\frac{1}{4}\left({\omega_{2}}^{2}-4\omega_{1}\omega_{2}\right)$$

The three differential forms from Equation (11) constitute a coframe [20] in any point of the absolute space. This allows the translation of the geometric properties of the absolute space to algebraic properties linked to differential Equation (10).

The simplest of these properties refer to dynamics on matrix geodesics, which are directly translated to statistical properties. In this case, the 1-forms $\omega_{1},\;\omega_{2},\;\omega_{3}$ are differentiated exactly in the same parameter the length τ of the geodesic arc. Along these geodesics, Equation (10) is transformed into a Riccati-type differential equation:

(14) $$\frac{dt}{d\tau }=P\left(t\right),\;\;P\left(t\right)=a_{1}t^{2}+2a_{2}t+a_{3}$$

Here, the parameters $a_{1}$, $a_{2}$, $a_{3}$ are constants that characterize a certain geodesic of the family.

For obvious reasons it is important to identify the most general solution of the differential Equation (14). Reference [21] offers a modern and pertinent method for the integrability of the Riccati differential equation. For the present work, it is sufficient to be noted that the relations:

(15) $$t_{0}=-\frac{a_{2}}{a_{1}}+\frac{i}{a_{1}}\Omega ,\;\;{\bar{t}}_{0}=-\frac{a_{2}}{a_{1}}-\frac{i}{a_{1}}\Omega ,\;\;\Omega^{2}=a_{3}a_{1}-{a_{2}}^{2},\;\;i=\sqrt{-1}$$

can be assimilated to the roots of the polynomial $P\left(t\right)$. As such, the homographic transformation needs to be performed first:

(16) $$z=\frac{t-t_{0}}{t-{\bar{t}}_{0}}\;$$

and now it is easy to see, by means of direct calculation, that z is a solution of the differential equation:

(17) $$\dot{z}=2i\Omega z,\;\;z\left(\tau \right)=z\left(0\right)e^{i\Omega \tau }\;$$

Therefore, if the initial condition $z\left(0\right)$ is conveniently expressed, it is possible to obtain the general solution of the differential Equation (14), by inversing the transformation (16), which implies:

(18) $$t=\frac{t_{0}+rexp\left[2i\Omega \left(\tau -\tau_{0}\right)\right]{\bar{t}}_{0}}{1+rexp\left[2i\Omega \left(\tau -\tau_{0}\right)\right]}\;$$

where r and $r_{0}$ are two real constants, specific to the solution.

By using the relations (15), it is possible to obtain the solution in real terms, i.e.,

(19) $$z=-\frac{a_{2}}{a_{1}}+\frac{\Omega }{a_{1}}\left[\frac{2r\sin\left[2\Omega \left(\tau -\tau_{0}\right)\right]}{1+r^{2}+2r\cos\left[2\Omega \left(\tau -\tau_{0}\right)\right]}+i\frac{1-r^{2}}{1+r^{2}+2r\cos\left[2\Omega \left(\tau -\tau_{0}\right)\right]}\right]$$

which highlights a self-modulation of Ω through the Stoler-type transformation [22], which leads to a complex form of this complex parameter. Moreover, if it is noted:

(20) r=coth s 

Equation (19) becomes:

(21) $$z=-\frac{a_{2}}{a_{1}}+\frac{\Omega }{a_{1}}h\;$$

where h has the expression

(22) $$h=-i\frac{\cosh\;s-exp\left[-2i\Omega \left(\tau -\tau_{n}\right)\right]\sinh\;s}{\cosh\;s+exp\left[-2i\Omega \left(\tau -\tau_{n}\right)\right]\sinh\;s}\;$$

In accordance with [18,19], this complex parameter operates as a harmonic mapping between the usual space (i.e., the measurement space) and the hyperbolic one.

**Figure 18:**
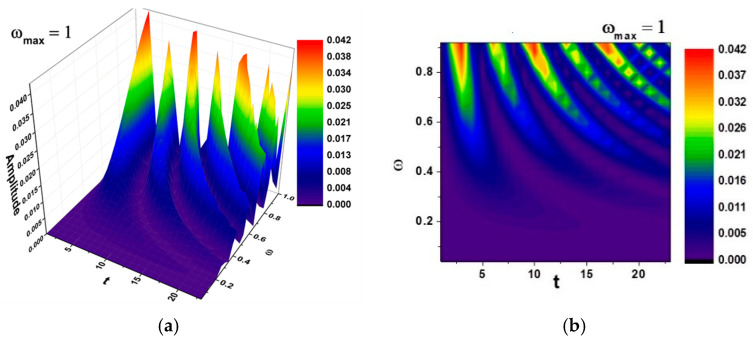
(**a**,**b**)—Three-dimensional and contour plot of the solution $Re\left(a_{1}z+a_{2}\right)\equiv Amplitude$ for the maximum value of the pulsation-type characteristic $\omega_{max}=1$ where Ω≡ω and t≡τ. Correspondence with Figure 16c is shown.

**Figure 19:**
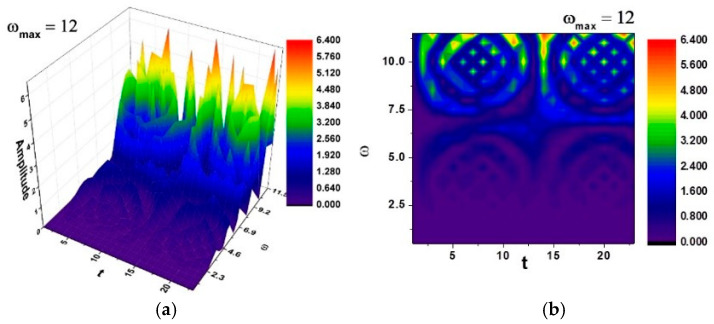
(**a**,**b**)—Three-dimensional and contour plot of the solution $Re\left(a_{1}z+a_{2}\right)\equiv Amplitude$ for the maximum value of the pulsation-type characteristic $\omega_{max}=12$ where Ω≡ω and t≡τ. Correspondence with Figure 14b is shown.

In Figure 18a,b and Figure 19a,b, self-modulations of the thermal field in forms that can “mimic” channel-type and cellular-type self-structures are presented [23,24], in correspondence with the experimentally obtained SEM analysis images.

Figure 18 and Figure 19 were generated using the previously-described boundary conditions, and the spatial modulation was fitted to a square sample domain of 500 × 500 µm, corresponding to the typical field-of-view of the SEM images. The initial perturbation was Gaussian, centered on the sample midplane, with an amplitude chosen to mimic thermal heterogeneity measured during mechanical cycling.

The theoretical patterning, channel-type in Figure 18 and cellular-type in Figure 19, provides an explanatory framework for the complex microstructures visualized in the SEM results (please see Figure 14, Figure 15 and Figure 16). The alignment between computed isothermal contours and martensitic plate orientations reinforces the physical plausibility of the proposed multifractal model.

**Figure 20:**
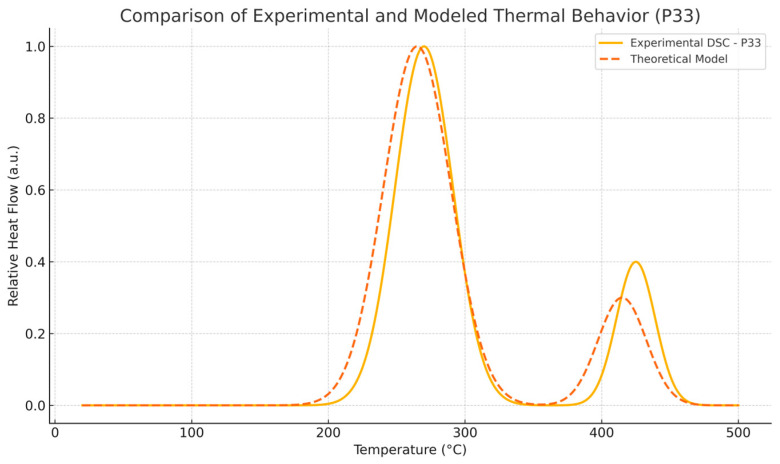
Overlay of modeled thermal profile and experimental DSC thermogram for sample P33, showing agreement in peak locations and profile shapes.

In addition, in Figure 20 found below, an overlaying of the modeled thermal front with a representative DSC curve for sample P33 offers visual confirmation of the predicted behaviors.

## 4. Conclusions

This study integrates experimental investigations and multifractal modeling in order to analyze the thermal and structural behavior of Cu-Zn-Al shape memory alloys. The DSC and DTA thermograms revealed characteristic transformation peaks, confirming both direct and inverse martensitic transitions and highlighting a recurrent endothermal event between 400 °C and 500 °C, linked to order–disorder phenomena.

The XRD analysis confirmed the co-existence of austenitic and martensitic phases across various compositions, while the SEM imaging revealed microstructural motifs (such as lamellar martensite and phase-separated dendrites), which are consistent with theoretical predictions of cellular and channel-type thermal modulations.

The multifractal thermal transfer model provided a robust framework to capture the non-linear, scale-dependent thermal behavior observed in the Cu-Zn-Al system. By incorporating experimental input parameters, the model was able to reproduce key features of the observed thermal dynamics, which are not adequately described by classical Fourier models.

Furthermore, the simulations of thermal patterning based on multifractal principles provided explanatory insight into the formation of complex microstructures observed in SEM analysis, establishing a theoretical–experimental link between thermal diffusion behavior and metallurgical phase morphology.

Future research will focus on quantitative calibration of the multifractal diffusivity function using in situ thermal imaging and on extending the model toward 3-D simulation frameworks. Additional techniques, such as EBSD and nanoindentation, will be employed, in order to assess the correlation between localized microstructure and macroscopic functional behavior. Moreover, unlike Fourier or Cattaneo–Vernotte models, which assume constant thermal properties, the multifractal approach accounts for evolving scale dependencies during phase transitions, offering deeper insight into SMA behavior.

## Figures and Tables

**Table 1:** Compositional data for Cu-Zn-Al alloy samples.

Sample No.	Cu (%)	Zn (%)	Al (%)	Trace Materials (%)
P5	65.45	20.71	11.72	0.05
P10	68.35	16.36	15.25	0.04
P13	67.36	18.16	14.45	0.03
P16	68.85	16.28	14.81	0.04
P20	72.22	13.56	14.19	0.03
P26	70.19	16.51	13.18	0.04
P30	68.81	19.45	11.69	0.05
P33	68.89	20.14	10.92	0.05
P36	69.87	17.52	12.57	0.04

**Table 2:** The identification of the type of network through the obtained peaks.

Pos. [°2Th.]	Maximum [cts]	FWHM [°2Th.]	Miller Index	Matching/Fitting	Material Structure	Lattice (Å) Parameter
39.68	146	0.38(3)	001	98-018-2360	ZnO—cubic	
40.893	361	0.45(3)	022	98-060-6887	AlCu_4_—cubic	
42.464	2841	0.269(3)	111	98-062-9465; 98-015-1371	Cu_2.8_Zn_1.2_—cubic	3.684
43.5909	15,790	0.345(1)	022	98-010-5026; 98-060-6887	Al_0.724_Cu_2.68_Zn_0.596_—cubic	5.866
44.034	3264	0.294(5)	033	98-015-1371	Al_4_Cu_9_—cubic	
45.779	493	0.71(3)	013	98-060-6887; 98-015-1371	AlCu_4_—cubic	6.261
49.401	646	0.44(1)	002	98-062-9465; 98-015-1371	Cu_2.8_Zn_1.2_—cubic	
63.405	412	0.72(3)	004	98-010-5026	Al_0.724_Cu_2.68_Zn_0.596_—cubic	
70.26	115	0.68(5)	233	98-060-6887	AlCu_4_—cubic	
72.83	190	1.17(4)	022	98-062-9465	Cu_2.8_Zn_1.2_—cubic	
77.555	189	0.38(2)	143	98-060-6887; 98-015-1371	AlCu_4_—cubic	

## Data Availability

The data presented in this study are available from the corresponding authors upon reasonable request.

## Appendix A

Let it be considered that the Cu-Zn-Al alloy can be viewed as a multifractal mathematical object, in both structure and function. The dynamics of such an object, as defined by the Multifractal Theory of Motion [1,2,3], are illustrated by continuous and non-differentiable curves (multifractal curves), which involves the functionality of the scale covariant derivative.

(A1) $$\frac{\hat{d}}{dt}=\partial t+{\hat{V}}^{e}\partial_{e}+D^{ie}\partial_{i}\partial_{e},\;\;i,e=1,2,3$$

where the subsequent notations are employed:

(A2)
$$\begin{array}{c} \partial_{t}=\frac{\partial }{\partial t},\;\;\partial_{e}=\frac{\partial }{\partial x^{e}},\;\;\partial_{i}\partial_{e}=\frac{\partial }{\partial x^{i}}\frac{\partial }{\partial x^{e}}\;\\ {\hat{V}}^{e}=V_{D}^{e}-iV_{F}^{e},\;\;i=\sqrt{-1}\\ D^{ie}={\frac{1}{4}\left(dt\right)}^{\left[\frac{2}{f\left(\alpha \right)}\right]-1}\left(d^{ie}+i{\bar{d}}^{ie}\right)\\ d^{ie}=\left(\lambda_{+}^{i}\lambda_{+}^{e}-\lambda_{-}^{i}\lambda_{-}^{e}\right),\;\;{\bar{d}}^{ie}=(\lambda_{+}^{i}\lambda_{+}^{e}+\lambda_{-}^{i}\lambda_{-}^{e}) \end{array}$$

In (A1) and (A2), $x^{e}$ defines the spatial coordinates, depicted by continuous and nondifferentiable mathematical functions that rely on the scale resolution. The temporal coordinate, *t*, is depicted by continuous and differentiable mathematical functions that do not rely on scale resolution. $V_{D}^{e}$ defines the differentiable velocity (i.e., the velocity at differential scale resolution), while $V_{F}^{e}$ illustrates the nondifferentiable velocity (i.e., the velocity at nondifferentiable scale resolution). $\lambda_{+}^{e}$ and $\lambda_{-}^{e}$ are particular constants of the differentiable–nondifferentiable scale transition coupled with the forward and backward SMA dynamics, respectively. The singularity spectrum of order α, identified through $f\left(\alpha \right)$, is defined by $\alpha =\alpha \left(D_{F}\right)$, where $D_{F}$ represents the fractal dimension of the motion curves of the structural units of any SMA. It is worth noting that the SMA is likened to a complex system at all times [1,2,3].

In this frame of reference, employing the singularity spectrum of order α to characterize SMA dynamics has evident advantages:

i. The dynamics pertaining to the structural units of the SMA may reveal a dominant fractal dimension, which may help in pinpointing a global SMA pattern which is consistent with a particular global SMA structurality and functionality.
ii. The dynamics pertaining to the structural units of the SMA could include a “collection” of fractal dimensions, which may help in pinpointing local SMA patterns that are compatible with particular, zone-specific SMA structures and functions.
iii. By means of the α-order singularity spectrum, one can pinpoint universality classes in the domain of SMA dynamics, even when the attractors related to said dynamics have distinct aspects.

The explicit configuration of the tensor $D^{ie}$, i.e., the one connected to the non-multifractal scale transition, is prescribed by multifractalization through stochasticity. A very usual procedure of fractalization is by means of stochastic processes [1,4,5]. In such a conjecture, it is essential to explain that the stochastic or chaotic processes employed in the study of SMA dynamics can be split into two categories. The first category comprises homogeneous SMA dynamics, which are described through a single global fractal dimension and display the identical scaling properties in any time interval (such as fractional Brownian processes). The second category entails SMA dynamics that can be depicted by employing multiple fractal dimensions and strictly quantifying local singularities, known as multifractal processes.

As such, it is possible to identify the subsequent types of SMA dynamics:

i. Multifractal SMA dynamics by means of Markov stochasticity, dictated through the subsequent boundaries [7,24]:

(A3) $$\lambda_{+}^{i}\lambda_{+}^{e}=\lambda_{-}^{i}\lambda_{-}^{e}=-2\lambda \delta^{ie}$$

where λ is a diffusion-type coefficient coupled with the multifractal-non-multifractal scale transition, and $\delta^{ie}$ is the Kronecker pseudotensor. Therefore:

(A4) $$D^{ie}\to i\lambda {\left(dt\right)}^{\left[\frac{2}{f\left(\alpha \right)}\right]-1}\delta^{ie}$$

in a way that Equation (A1) emerges as below:

(A5) $$\frac{\hat{d}}{dt}=\partial_{t}+{\hat{V}}^{e}\partial_{e}-i\lambda {\left(dt\right)}^{\left[\frac{2}{f\left(\alpha \right)}\right]-1}\partial^{e}\partial_{e}$$

ii. Multifractal SMA dynamics through non-Markov stochasticity dictated by the subsequent boundaries [3]:

(A6) $$d^{ie}=4\alpha \delta^{ie},\;\;{\bar{d}}^{ie}=-4{\beta \delta }^{ie}\;$$

where α and β are diffusion-type coefficients coupled with the multifractal-non-multifractal scale transition. As such, the following case results:

(A7) $$D^{ie}=\left(\alpha -i\beta \right){\left(dt\right)}^{\left[\frac{2}{f\left(\alpha \right)}\right]-1}\delta^{ie}$$

in a way that Equation (A1) emerges as such:

(A8) $$\frac{\hat{d}}{dt}=\partial_{t}+{\hat{V}}^{e}\partial_{e}-(\alpha -i\beta )\;{\left(dt\right)}^{\left[\frac{2}{f\left(\alpha \right)}\right]-1}\partial^{e}\partial_{e}$$

Now, presuming the functionality of the principle of scale covariance, based on which the laws of SMA physics stay invariant regarding both spatial and temporal transformations and scale transformations, various conservation laws can be built.

For instance, if one utilizes the complex operator (A8) upon the temperature field of any complex system, then the conservation law has the shape:

(A9) $$\frac{\hat{dT}}{dt}=\partial_{t}T+{\hat{V}}^{e}\partial_{e}T-\left(\alpha -i\beta \right){\left(dt\right)}^{\left[\frac{2}{f\left(\alpha \right)}\right]-1}\partial^{e}\partial_{e}T=0$$

or described on resolution scales as such:

(A10) $$\partial_{t}T+V_{D}^{e}\partial_{e}T+\alpha \;{\left(dt\right)}^{\left[\frac{2}{f\left(\alpha \right)}\right]-1}\partial^{e}\partial_{e}T=0$$

at differential scale resolutions, i.e., as below:

(A11) $$-V_{F}^{e}\partial T-\beta \;{\left(dt\right)}^{\left[\frac{2}{f\left(\alpha \right)}\right]-1}\partial^{e}\partial_{e}T=0$$

at non-differential scale resolutions. In the following, if the following notation is used:

(A12) $$V^{e}=V_{D}^{e}-V_{F}^{e}$$

the velocity related to the multifractal-non-multifractal scale transition, by summing up relations (A10) and (A11), the conservation law of the temperature field affiliated with the multifractal-non-multifractal scale transition is extracted as follows:

(A13) $$\partial_{t}T+V^{e}\partial_{e}T+(\alpha -\beta )\;{\left(dt\right)}^{\left[\frac{2}{f\left(\alpha \right)}\right]-1}\partial^{e}\partial_{e}T=0$$

In what follows, the consequences of this type of differential equation in modelling SMA dynamics are presented. For this reason, let the differential Equation (A13) in the one-dimensional case be considered and subjected to the conditions below:

(A14) $$V^{e}\equiv (0,0,V=const)$$

This then becomes:

(A15) $$\frac{\partial T}{\partial t}+V\frac{\partial T}{\partial z}-D\frac{\partial^{2}T}{\partial z^{2}}=0$$

where the notation is made:

(A16) $$D=(\beta -\alpha )\;{\left(dt\right)}^{\left[\frac{2}{f\left(\alpha \right)}\right]-1}$$

where *D* depends both on the structural properties and scale resolution scale properties of the SMA.

The above model can describe, for example, at various scale resolutions, nonlinear behaviors of any SMA that undergo transformations of state. From this perspective, the multifractal–nonmultifractal scale transition can be assimilated to order–disorder-type transition, where V denotes the propagation speed of the phase transformation associated with such transition.

Through the variable change below:

(A17) $$T\left(z,t\right)=\theta \left(z,t\right)\exp(\frac{Vz}{2D}-\frac{V^{2}t}{4D})$$

which implies that:

(A18) $$\begin{array}{c} \frac{\partial T}{\partial t}=(\frac{\partial \theta }{\partial t}-\frac{V^{2}\theta }{4D})exp(\frac{Vz}{2D}-\frac{V^{2}t}{4D})\\ \frac{\partial T}{\partial z}=(\frac{\partial \theta }{\partial z}+\frac{V\theta }{2D})exp(\frac{Vz}{2D}-\frac{V^{2}t}{4D})\\ \frac{\partial^{2}T}{\partial z^{2}}=(\frac{\partial^{2}\theta }{\partial z^{2}}+\frac{V}{D\;}\;\frac{\partial \theta }{\partial z}+\frac{V^{2}\theta }{4D^{2}})exp(\frac{Vz}{2D}-\frac{V^{2}t}{4D}) \end{array}$$

the differential Equation (A15) is then:

(A19) $$\frac{\partial \theta }{\partial t}=D\frac{\partial^{2}\theta }{\partial z^{2}}$$

On the basis of the initial conditions and the boundary one:

(A20) $$\begin{array}{c} T\left(z,0\right)=T_{0}=const\\ T\left(0,t\right)=0 \end{array}$$

the boundary conditions associated to θ are extracted as below:

(A21) $$\begin{array}{c} \theta \left(z,0\right)=\theta_{0}exp(\frac{Vz}{2D})\\ \theta \left(0,t\right)=0 \end{array}$$

This issue can be restricted to one devoid of boundary conditions by expanding the solution to take into account the region z<0, where θ presumes an original distribution which mimics the boundary condition for z=0 at any time t>0. Simultaneously, it is possible to select that:

(A22) $$\theta \left(z,0\right)=\theta (-z,0)$$

Thus, for any expanded domain, the initial condition is stated as:

(A23) $$\theta \left(z,0\right)=\left\{\begin{array}{c} \theta_{0}\exp\left(\frac{Vz}{2D}\right),\;z>0\\ -\theta_{0}\exp\left(-\frac{Vz}{2D}\right),\;z<0 \end{array}\right.$$

The issue now is how to “work out” the temperature equation for an originally stated dimensional distribution in an unbounded medium. Such an issue can be addressed by constructing the solution as a Fourier integral:

(A24) $$\theta \left(z,t\right)=\int_{-\infty }^{+\infty }\phi \left(k,t\right)\exp\left(ikz\right)dk$$

(A25) $$\phi \left(k,t\right)=\frac{1}{2\pi }\int_{-\infty }^{+\infty }\theta (z^{\prime },t)\exp\left(-ikz^{\prime }\right)dz\prime$$

As soon as the differential Equation (A19) is replaced, it is obtained:

(A26) $$\frac{\partial \phi }{\partial t}+k^{2}D\phi =0$$

having the solution:

(A27) $$\phi \left(k,t\right)=\phi \left(k,0\right)exp(-k^{2}Dt)$$

where $\phi \left(k,0\right)$ was extracted by supplanting the initial distribution *n* into Equation (A25). As such, Equation (A24) can be stated as an integral across the whole initial distribution:

(A28) $$\theta \left(z,t\right)=\frac{1}{2\pi }\int_{-\infty }^{+\infty }\int_{-\infty }^{+\infty }\theta (z^{\prime },0)\exp\left(-k^{2}Dt\right)exp\left[ik(z-z^{\prime })\right]dz\prime dk$$

As soon as the aforementioned integral is resolved concerning *k*, computing it for *n* becomes simple, pointing towards:

(A29) $$\theta \left(z,t\right)=\frac{1}{{\left(4\pi Dt\right)}^{\frac{1}{2}}}\int_{-\infty }^{+\infty }\theta (z^{\prime },0)\exp\left[-\frac{{\left(z-z^{\prime }\right)}^{2}}{4Dt}\right]dz\prime$$

The solution can be concluded through substituting Equation (A23) into Equation (A29) and computing the integral. The result for $T\left(z,t\right)$ is granted by:

(A30) $$T\left(z,t\right)=\frac{\theta_{0}}{2}\exp\left(-\frac{V^{z}t}{D}\right)+\frac{\theta_{0}}{2}\exp\left(-\frac{V^{z}t}{D}\right)\cdot \left[\frac{erf(z-Vt)}{{\left(4Dt\right)}^{\frac{1}{2}}}+\frac{erf(z+Vt)}{{\left(4Dt\right)}^{\frac{1}{2}}}\right]$$

where erf(x) is the Laplace function imparted by:

(A31) $$\mathrm{erf}\left(x\right)=\frac{1}{2\sqrt{\pi }}\int_{0}^{\infty }\exp\left(-x^{2}\right)dx$$

values of this function being tabulated.

The expansion velocity of the thermal field on the surface z=0 is given through:

(A32) $$\hat{V}=\frac{V}{2}\cdot \left[1+\mathrm{erf}{\left(\frac{V^{2}t}{4D}\right)}^{\frac{1}{2}}\right]+{\left(\frac{D\theta_{0}}{\theta t}\right)}^{\frac{1}{2}}exp\left(-\frac{V^{2}t}{4D}\right)$$
